# The Role of MicroRNAs in Mitochondria-Mediated Eye Diseases

**DOI:** 10.3389/fcell.2021.653522

**Published:** 2021-06-18

**Authors:** Sabrina Carrella, Filomena Massa, Alessia Indrieri

**Affiliations:** ^1^Telethon Institute of Genetics and Medicine, Naples, Italy; ^2^Institute for Genetic and Biomedical Research, National Research Council (CNR), Milan, Italy

**Keywords:** microRNA, retina, mitochondria, mitochondrial diseases, glaucoma, AMD, diabetic retinopathy, MitomiR

## Abstract

The retina is among the most metabolically active tissues with high-energy demands. The peculiar distribution of mitochondria in cells of retinal layers is necessary to assure the appropriate energy supply for the transmission of the light signal. Photoreceptor cells (PRs), retinal pigment epithelium (RPE), and retinal ganglion cells (RGCs) present a great concentration of mitochondria, which makes them particularly sensitive to mitochondrial dysfunction. To date, visual loss has been extensively correlated to defective mitochondrial functions. Many mitochondrial diseases (MDs) show indeed neuro-ophthalmic manifestations, including retinal and optic nerve phenotypes. Moreover, abnormal mitochondrial functions are frequently found in the most common retinal pathologies, i.e., glaucoma, age-related macular degeneration (AMD), and diabetic retinopathy (DR), that share clinical similarities with the hereditary primary MDs. MicroRNAs (miRNAs) are established as key regulators of several developmental, physiological, and pathological processes. Dysregulated miRNA expression profiles in retinal degeneration models and in patients underline the potentiality of miRNA modulation as a possible gene/mutation-independent strategy in retinal diseases and highlight their promising role as disease predictive or prognostic biomarkers. In this review, we will summarize the current knowledge about the participation of miRNAs in both rare and common mitochondria-mediated eye diseases. Definitely, given the involvement of miRNAs in retina pathologies and therapy as well as their use as molecular biomarkers, they represent a determining target for clinical applications.

## Introduction

Mitochondria are key players in different cellular processes, and their dysfunction contributes to the pathogenesis of neurodegenerative disorders (NDs), including many retinal diseases. To date, a connection between vision and defective mitochondrial functions has been extensively described (Yu-Wai-Man et al., 2011; Gueven et al., 2017). Mitochondrial diseases (MDs) are a heterogeneous group of rare disorders caused by mutations in nuclear or mitochondrial genes that affect proteins essential for mitochondrial structure and function. Although they are highly genetically and clinically heterogeneous, several MDs, such as Leber hereditary optic neuropathy (LHON), autosomal dominant optic atrophy (ADOA), and neuropathy, ataxia, and retinitis pigmentosa (NARP), show some form of vision impairment and can be classified as primary mitochondrial eye diseases (PMEDs). Moreover, mitochondrial dysfunctions represent a common denominator and a common cause of neuronal death involved in the pathogenesis of many NDs due to mutations in genes encoding non-mitochondrial proteins or characterized by more complex pathogenetic events (Niyazov et al., 2016).

**FIGURE 1:**
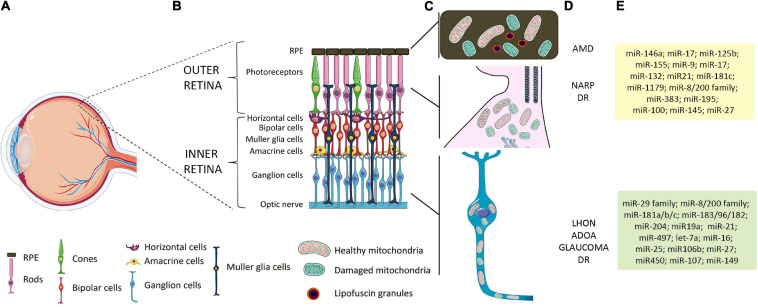
Schematic representation of retinal layers, mitochondrial distribution, and related retinal pathologies and miRNAs. **(A)** Schematic representation of the human eye. The box identifies the position of the retinal layers in the posterior part of the eye. **(B)** Magnification of the retina and retinal layers in healthy conditions. **(C)** A peculiar distribution of mitochondria is graphically represented: in RPE, which overlays the PRs, mitochondria are located at the basal region; in PRs, a high number of mitochondria are present in the inner segment; in the inner retina, mitochondria are predominantly concentrated in the unmyelinated proximal axons of RGCs. **(D)** Primary or secondary defect in mitochondrial functionality in RPE and PRs has been linked to AMD, NARP, and other forms of visual loss, such as DR. Primary or secondary impairment in mitochondrial function leading to RGC degeneration is considered a major cause of retinal diseases such as LHON, ADOA, glaucoma, and DR. **(E)** List of miRNAs expressed in specific cell retinal layers whose function or alteration in expression has been linked to mitochondrial dysfunction and retinal diseases. Yellow box shows relevant miRNAs related to AMD and DR; green box, LHON and glaucoma-related miRNAs. RPE, retinal pigment epithelium; PRs, photoreceptors; RGCs, retinal ganglion cells; AMD, age-related macular degeneration; NARP, neuropathy, ataxia, and retinitis pigmentosa; DR, diabetic retinopathy; LHON, Leber hereditary optic neuropathy; ADOA, autosomal dominant optic atrophy.

The great concentration of mitochondria in metabolically active tissues with high-energy demands, such as the retina, makes them particularly sensitive to mitochondrial dysfunction. The retina comprises different cell types organized in layers that form neuronal circuits working in parallel and in combination to produce a complex visual output (Figure 1) (Carrella et al., 2020). The outer nuclear layer (ONL) is composed of photoreceptor cells (PRs), subdivided into rods and cones. They synapse with interneurons of the inner nuclear layer (INL), namely, bipolar cells, amacrine cells, and horizontal cells, which in turn contact RGCs in the RGC layer. Retinal layers show a peculiar distribution of mitochondria to guarantee the energy supply for the conversion and propagation of the light signal (Figure 1). PRs, which capture photons and generate electrophysiological signals, display many mitochondria in the inner segment. In RPE, mitochondria are located at the basal region, that is, in contact with PRs. Instead, in the inner retina, mitochondria are predominantly concentrated in the unmyelinated proximal axons of RGCs, which transmit visual information to the brain. It is thus not surprising that the most common retinal disorders, i.e., glaucoma, age-related macular degeneration (AMD), and diabetic retinopathy (DR), show mitochondrial dysfunction and share some clinical similarities with PMEDs (Carelli et al., 2004; Yu-Wai-Man et al., 2011; Gueven et al., 2017; Ferrington et al., 2020). Interestingly, many studies also reported vision impairment and retinal abnormalities in the majority of Alzheimer’s and Parkinson’s disease patients and animal models, highlighting the involvement of mitochondrial anomalies in the development of visual defects (Colligris et al., 2018; Indrieri et al., 2020b; Marrocco et al., 2020; Mirzaei et al., 2020).

MicroRNAs (miRNAs) are a class of non-coding RNAs able to post-transcriptionally regulate gene expression through a powerful mechanism of sequence-specific recognition. Each miRNA is predicted to recognize about 200 mRNA targets, guaranteeing a pleiotropic fine-tuning of correlated transcripts that confers robustness to pathway regulation (Bartel, 2018).

Soon, their relevant role in different retina pathologies (Karali and Banfi, 2019; Zuzic et al., 2019) and the possibility to exploit their modulation as a possible gene/mutation-independent strategy for these disorders became evident (Carrella et al., 2020). The extensive genetic heterogeneity of many inherited retinal disorders, including PMEDs, indeed represents a significant limitation to the development and application of gene-replacement therapy in most of patients. Moreover, gene replacement cannot be applied in disorders caused by gain-of-function mutations and too complex multifactorial diseases such as AMD, glaucoma, and DR (Carrella et al., 2020). In this respect, miRNAs represent interesting therapeutic targets able to regulate common dysregulated pathways underlying retinal damage.

Moreover, dysregulated miRNA expression profiles in retinal degeneration models and in patients indicate that they may represent reliable biomarkers for the diagnosis of these disorders or to predict the onset and the progression of the disease, and the evaluation of the response to treatments. Circulating miRNAs and exosomal miRNAs can be indeed easily detected, thus representing promising disease predictive/diagnostic/prognostic biomarkers (Saxena et al., 2015; Palfi et al., 2016; Anasagasti et al., 2018).

MicroRNAs can localize to different subcellular compartments (i.e., mitochondria, endoplasmic reticulum, and exosomes) (Leung, 2015); and an increasing interest is growing about miRNAs, called MitomiRs, that regulate mitochondrial function. MitomiRs can be divided into two subgroups: those binding to nuclear-transcribed mRNA encoding mitochondrial proteins and those imported into mitochondria targeting mitochondrial-encoded mRNAs (Purohit and Saini, 2021). Moreover, some MitomiRs (i.e., miR-1974, miR-1977, and miR-1978) may be transcribed by the mitochondrial DNA (mtDNA) (Bandiera et al., 2011); however, more data are necessary to validate these findings.

Modulation of miRNAs has been recently applied as therapy to different disorders reaching preclinical and clinical stages (Bajan and Hutvagner, 2020). However, investigations on the role of miRNAs, and specifically MitomiRs, in mitochondrial-mediated disorders are few. In this review, we will summarize the current knowledge about the involvement of miRNAs in mitochondria-mediated eye diseases, including both rare PMEDs and the most common retinal disorders, i.e., glaucoma, AMD, and DR. In particular, their role in retina pathologies and therapy, as well as their role as biomarkers in these disorders, will be analyzed, highlighting their huge potential in clinical medicine.

## Mitochondria-Mediated Eye Diseases

### Primary Mitochondrial Eye Diseases

#### Leber Hereditary Optic Neuropathy

Leber hereditary optic neuropathy is one of the most frequent PMEDs with a prevalence of between 1/15,000 and 1/50,000 people worldwide. LHON is an organ-specific disease, characterized by death of RGCs leading to degeneration of the optic nerve (ON) and bilateral or unilateral loss of vision, which typically occurs between the ages of 20 and 40 (Meyerson et al., 2015). It shows maternal inheritance, and it results more commonly in men, with variable disease penetrance. Approximately 95% of LHON cases are associated with three mtDNA point mutations (m.11778G > A, m.3460G > A, and m.14484T > C) that primarily affect mitochondrial respiratory chain (MRC) complex I genes (*ND1*, *ND4*, and *ND6*) (Yu-Wai-Man et al., 2002; Newman, 2005).

The molecular mechanism underlying death of RGCs is still not clear, even if it has been correlated to a reduction of ATP, an increase of reactive oxygen species (ROS) production due to defective MRC, and a significantly impaired mitophagy (Sharma et al., 2019).

#### Autosomal Dominant Optic Atrophy

With a prevalence of 1/10,000–1/35,000, ADOA is the most common form of PMEDs due to nuclear DNA mutations. Bilaterally symmetric progressive deterioration of the central visual acuity, ON pallor, dyschromatopsia, and blindness are the main symptoms, usually beginning in childhood (Fraser et al., 2010; Yu-Wai-Man et al., 2014). As in LHON, the disease primarily affects the RGCs and their axons, even if the ADOA progression with age is highly variable (Lenaers et al., 2012). In about 50–60% of the cases, patients harbor mutations in the *OPA1* (Alexander et al., 2000; Ferre et al., 2005). In addition, other mutated genes include *OPA2*, *OPA3*, *OPA4*, *OPA5*, *OPA8*, *WFS1*, and *SSBP1* (Finsterer et al., 2018; Piro-Megy et al., 2020).

OPA1 is a crucial component of the mitochondrial fusion machinery and also controls crista biogenesis and remodeling, impacting apoptosis and mitochondrial respiration (Cogliati et al., 2016). In accordance, Opa1 deficiency induces a significant fragmentation of the mitochondrial network and impairs ON structure and visual function in a mouse model of ADOA (Davies et al., 2007).

Recently, the role of autophagy in the regulation of mitochondrial distribution in axons of RGC and in visual loss in an ADOA mouse model (Zaninello et al., 2020) has also been demonstrated, indicating an important patho-mechanism contribution of mitophagy.

#### Neuropathy, Ataxia, and Retinitis Pigmentosa

Neuropathy, ataxia, and retinitis pigmentosa is an inherited neurologic/metabolic syndrome whose clinical hallmarks are (i) sensory neuropathy including progressive motor weakness and lethargy, (ii) ataxia, which affects the balance and coordination, and (iii) ophthalmologic findings including retinitis pigmentosa, optic atrophy, and eye movement disorders. Usually, the retina defects worsen over time, leading to severe vision loss and blindness. NARP typically begins in childhood or early adulthood. The clinical expression of the NARP syndrome is very variable, and the predominant ocular manifestation is characterized by an initial RPE degeneration and a rod/cone dysfunction in different families (Gelfand et al., 2011).

neuropathy, ataxia, and retinitis pigmentosa results from mtDNA heteroplasmic mutations in *ATP6* gene (predominantly m.8993T > G/C), coding for the mitochondrial ATP synthase subunit 6 (Holt et al., 1990; Duno et al., 2013; Miyawaki et al., 2015). ATP synthase impairment affects oxidative phosphorylation, causing energy deprivation and overproduction of ROS (Nijtmans et al., 2001; Baracca et al., 2007).

### Eye Diseases Associated With Mitochondrial Dysfunctions

#### Glaucoma

With about 70 million patients worldwide, glaucoma is the leading cause of blindness and a major economic burden (Quigley and Broman, 2006). The term glaucoma describes a group of complex multifactorial diseases characterized by ON damage and loss of RGCs resulting in progressive loss of vision. Age, genetics, and elevated intraocular pressure (IOP) are prominent risk factors; however, about one-third of cases have ON degeneration despite IOP in the normal range (Almasieh and Levin, 2017).

Interestingly, similarities between glaucoma and PMEDs have been described, and defects in mitochondria have been connected to glaucomatous neurodegeneration (Kong et al., 2009; Sundaresan et al., 2015; Shim et al., 2016; Williams et al., 2017; Singh et al., 2018; Tribble et al., 2019). Moreover, mutations in *OPTN* encoding for the mitophagy adaptor protein optineurin (Rezaie et al., 2002; Wong and Holzbaur, 2014), *TBK1* encoding the serine threonine protein kinase TANK-binding kinase 1 involved in autophagy (Sears et al., 2019), and *OPA1* (Aung et al., 2002; Yu-Wai-Man et al., 2010; Guo et al., 2012) have been associated with glaucoma, thus highlighting a crucial role for mitochondrial dynamics and mitophagy pathways in glaucoma pathogenesis (Ito and Di Polo, 2017). In addition, numerous studies reported mtDNA mutations, decrease in the mitochondrial respiratory activity, and oxidative stress in glaucoma patients and in animal models of this disease (Lee et al., 2011; Tseng et al., 2015; Hondur et al., 2017; Williams et al., 2017; Tribble et al., 2019). Notably, mitochondrial dysfunction can be detected before RGC death occurs in glaucoma animal models (Kong et al., 2009; Williams et al., 2017; Singh et al., 2018), suggesting a primary effect for mitochondrial abnormalities in glaucoma onset.

#### Age-Related Macular Degeneration

Mitochondrial dysfunction has been implicated in the pathophysiology of several age-related diseases including those that involve PRs and RPE cells (Lukiw et al., 2012; Lefevere et al., 2017; Ferrington et al., 2020). Aging and oxidative stress have been recognized as primary risk factors for AMD (Liang and Godley, 2003; Jarrett and Boulton, 2012; Lefevere et al., 2017), a complex degenerative and progressive disease.

There are two forms of AMD: the “wet” form that is associated with abnormal growth of blood vessels into the retina and the “dry” form with primary pathogenic event involving RPE degeneration causing PR cell death (Liang and Godley, 2003). RPE cells engulf photoreceptor outer segments (POSs) that are shed daily during renewal of PRs. RPE accumulation of lipofuscin, a product of POS turnover, has been hypothesized to be the primary source of ROS responsible for oxidative damage of the RPE resulting in impaired metabolism and apoptosis (Liang and Godley, 2003; Vives-Bauza et al., 2008; Jarrett and Boulton, 2012). Several studies have provided evidence that impaired autophagy (Mitter et al., 2014; Hyttinen et al., 2017) and mitochondrial dysfunction (Barron et al., 2001; Feher et al., 2006), in both RPE and PRs, exacerbate oxidative stress and contribute to the pathogenesis of AMD.

#### Diabetic Retinopathy

Diabetic retinopathy represents one of the most common slow-progressing microvascular complications of diabetes. In diabetic patients, damaged blood vessel of the retina leads to retinal detachment and reduction in the visual field and blindness (Frank, 2004). The retinal neurodegeneration is associated with retinal electrophysiological dysfunction and thinning of RGC and PR layers (Carbonell et al., 2019). Accelerated apoptosis of both neuronal and vascular cells (Mizutani et al., 1996; Barber et al., 2011) indicates apoptotic cell death as a contributing process to DR.

Although the detailed mechanisms of action in the development of DR are still unknown, involvement of mitochondrial dysfunctions with ROS formation and a decrease of the mitochondrial fusion protein mitofusin 2 (Mfn2) have been found in experimental models of this retinopathy (Eshaq et al., 2014; Duraisamy et al., 2019).

## MicroRNAs in Mitochondria-Mediated Eye Diseases

As reported before, an increasing number of miRNAs have been shown to be involved in the regulation of mitochondrial metabolism, although there is no evidence, to date, that mitochondrial disorders affect their expression or are directly caused by their dysregulation. Recently, miR-181a and miR-181b (miR-181a/b) were shown to directly target genes involved in mitochondrial biogenesis and function, and ROS detoxification (Indrieri et al., 2019). Inactivation of miR-181a/b leads to increased levels of mitochondrial biogenesis and mitophagy leading to a significant amelioration of the disease phenotype in LHON mouse models. These data suggest that miR−181a/b may represent gene−independent therapeutic targets for mitochondrial-related eye diseases (Indrieri et al., 2019). In accordance with the pervasive and pleiotropic roles of the miR-181 family (Indrieri et al., 2020a), miR-181c might be associated with vascular proliferation in high glucose diabetic-like environment (Qing et al., 2014; Zitman-Gal et al., 2014).

Large-scale studies have been performed to identify glaucoma-relevant miRNAs (Li et al., 2009; Liu et al., 2018; Hindle et al., 2019). Among the 159 miRNAs identified, many were differentially expressed in the aqueous humor (AH) and/or tear of glaucoma patients and controls. MiRNA-29 family controls extracellular matrix (ECM) homeostasis in trabecular meshwork (TM) cells, by negatively regulating collagens, fibrillins, and elastin (Luna et al., 2009; Villarreal et al., 2011). Moreover, a specific crosstalk between TGFβ, whose alteration are often observed in glaucoma, and miR-29 levels highlighted miR-29-family implication in glaucoma (Luna et al., 2011). The expression profile of miR-8/miR-200 family is upregulated in transgenic mice carrying a mutation in *OPTN* (Chi et al., 2010; Gao et al., 2016). Moreover, miR-200c can decrease trabecular contraction and IOP by regulating genes associated with TM cell contraction regulation (Luna et al., 2012). The miR-183/96/182 cluster is highly expressed in retina and implicated in several aspects of retinal cell development and maintenance (Amini-Farsani and Asgharzade, 2020). In particular, miR-182 was found to be the most abundant miRNA also in the axons of developing RGC where it regulates axon guidance (Bellon et al., 2017). Interestingly, a case–control study conducted on patients with primary open-angle glaucoma (POAG) concludes that the carriers of polymorphism in miR-182 and *CDKN2B* genes have an increased risk of developing POAG (Moschos et al., 2020). MiR-204 caused reduced expression of *FOXC1*, implicated in glaucoma development, and its target genes (Paylakhi et al., 2013). Moreover, it has been shown that in ON injury, miR-204 can downregulate *GAP43*, which plays an important role in axonal growth and in experimental chronic glaucomatous injury (Huang et al., 2013; Wang et al., 2018). Moreover, overexpression of miR-19a augments axon regeneration via miR-19a–PTEN axis, underscoring the therapeutic potential of local administration of miRNAs via intravitreal injection (Mak et al., 2020). Another interesting miRNA is miR-21, whose inhibition in a model of ON crush promotes axonal regeneration and RGC survival and function (Li H. J. et al., 2018; Li et al., 2019). In the retina of rats with advanced nerve damage induced by elevated IOP, eight miRNAs were significantly downregulated as compared with those in controls (miR-181c, miR-497, miR-204, let-7a, miR-29b, miR-16, miR-106b, and miR-25) and miR-27a was significantly upregulated. Observed miRNA level alterations caused enrichment of targets associated with ECM/cell proliferation, immune system, and regulation of apoptosis (Jayaram et al., 2015). Several miRNAs have been also found to be released in extracellular space in glaucomatous AH. Released miRNAs include miR-21 (apoptosis), miR-450 (cell aging and maintenance of contractile tone), miR-107 (nestin expression and apoptosis), and miR-149 (endothelia and ECM homeostasis) (Tanaka et al., 2014; Izzotti et al., 2015).

Few dysregulated miRNAs in multiple studies have been identified in the blood and vitreous humor of AMD patients. The serum profiles of patients with both wet and dry AMD have shown differences and partial overlap in several miRNAs (Szemraj et al., 2015; Berber et al., 2017), reflecting the difficulty of reducing biomarkers for AMD to one common group (Natoli and Fernando, 2018). A group of dysregulated miRNAs were reported in mouse models of distinct AMD features and demonstrated some similarities with the human AMD findings, including miR-146a, miR-9, miR-17, miR-125b, and miR-155 (Lukiw et al., 2012; Berber et al., 2017; Natoli and Fernando, 2018; Pogue and Lukiw, 2018; Martinez and Peplow, 2021). Those miRNAs can be considered as potential biomarkers and as possible therapeutic targets for AMD. MiR-146a has been found in the plasma (Ertekin et al., 2014; Menard et al., 2016) and retinas (Bhattacharjee et al., 2016) of AMD patients and was modulated in human monocytes stimulated with lipopolysaccharide (Taganov et al., 2006). MiR-146a and miR-9 are upregulated by NF-κB and present indirect correlation with complement factor H (CFH) levels, a key repressor of the innate immune response and a key player in AMD pathogenesis, indicating their modulation as a therapeutic strategy (Lukiw et al., 2012). MiR-17, a regulator of angiogenesis (Doebele et al., 2010) and anti-apoptotic genes as well (Song et al., 2015), is upregulated in an oxidative-induced retina model, an oxidative stress model in RPE cells, and neovascularization AMD plasma. MiR-155 has a role in angiogenesis, complement activation, and inflammation, making it a candidate for therapeutic interventions for AMD. The expression of miR-155 is also induced by AMD-related inflammatory cytokines (O’Connell et al., 2007). In an animal model of AMD, miR-155 has been shown to be upregulated in correlation with increased cell death and inflammation (Saxena et al., 2015), and its downregulation reduced retinal neovascularization (Zhuang et al., 2015). In addition, miR-155 depletion correlates with decreased levels of the mitochondrial translocator protein (TSPO), a selective marker of microglia in their highly reactive state (Yan et al., 2015). Interestingly, miR-146a and miR-155 recognize an overlapping 3′ UTR in CFH, to which both miRNAs may interact (Lukiw et al., 2012).

Several miRNAs, related to DR, are involved in vasculature regulation (miR-126, miR-200b, and miR-31), chronic inflammation pathway (miR-146, miR-155, miR-132, and miR-21), and oxidative stress (miR-21, miR-181c, miR-1179, and miR-8/miR-200 family); other miRNAs present altered expression in DR, but their role is not yet defined (Wu et al., 2012; Andreeva and Cooper, 2014; Mastropasqua et al., 2014; Pusparajah et al., 2016; Shafabakhsh et al., 2019). MiR-383 presents an increased expression in hyperglycemic conditions and targets the mitochondrial peroxiredoxin 3 involved in ROS detoxification and apoptosis (Li et al., 2013). Indeed, miR-383 inhibition diminished ROS and cell death in RPE treated with high glucose (Jiang et al., 2017), representing one of the major keys for the treatment of DR. The expression of miR-451a was found downregulated in diabetic conditions. MiR-451a mimic overexpression showed a protective effect on mitochondrial function in diabetic conditions, probably via the downregulation of activating transcription factor 2 (ATF2) and its downstream target genes CyclinA1, CyclinD1, and MMP2, providing new perspectives for developing effective therapies for proliferative DR (Shao et al., 2019). In both experimental and human diabetes, miR-34a showed increased expression. It promotes mitochondrial dysfunction and retinal microvascular endothelial cell senescence by suppressing the SIRT1–PGC-1α axis as well as the mitochondrial antioxidants TrxR2 and SOD2 (Thounaojam et al., 2019). MiR-195 acts as a regulator for *Mfn2*, which is reduced in the retina of diabetic patients and is involved in maintaining mitochondrial morphology, fusion, and ROS metabolism (Sugioka et al., 2004; Zheng and Xiao, 2010). Oxidative stress-induced overexpression of miR-195 can result in the downregulation of Mfn2 leading to tube formation and to increased blood–retinal barrier permeability, which are two common pathogenic events of DR (Zhang et al., 2017). Therefore, miR-195 could be considered as a potential therapeutic target for DR (Zhong and Kowluru, 2011). Another miRNA increased upon oxidative stress is miR-100, able to downregulate AKT pathway, extracellular-signal regulated kinase pathway, and TrkB pathway (Kong et al., 2014). MiR-145 overexpression reduced ROS production and increased the activity of SOD (Hui and Yin, 2018). Finally, miR-27 reduces ROS generation and downregulates the P13K/AKT/mTOR signaling pathway by inhibition of Nox2 (Li J. et al., 2018) implicated in ROS induction and neovascularization (Chan et al., 2013, 2015).

Overall, the positive effect of miR-19a, miR-204, and miR-21 modulation on glaucoma murine models, as well as downregulation of miR-155 in AMD mice, highlights the possibility of their rapid translation into clinical application as therapeutic molecules for these eye diseases (bold miRNAs in Table 1). However, other preclinical validation steps are required for most of the previously mentioned miRNAs, thus underlining the need and importance of this emerging field of research.

**TABLE 1:**
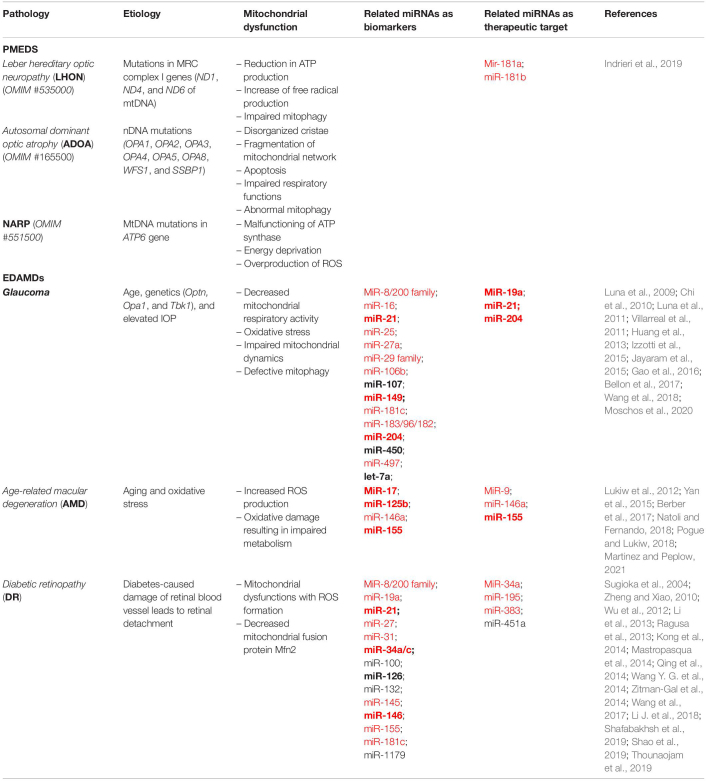
Summary of miRNAs involved in mitochondria-mediated eye diseases.

*MitomiRs are highlighted in red. The most promising miRNAs for clinical development are in bold. mtDNA, mitochondrial DNA; ATP, adenosine triphosphate; nDNA, nuclear DNA; ROS, reactive oxygen species; IOP, intraocular pressure; Mfn2, mitofusin 2; PMEDs, primary mitochondrial eye diseases; NARP, neuropathy, ataxia, and retinitis pigmentosa; EDAMDs, eye diseases associated with mitochondria dysfunctions.*

Systematic expression profiling of miRNAs in retinal cells could be of benefit to identify possible involvement of their function in specific retinal cell types, in physiological and pathological conditions. Although novel strategies are under development to study miRNA expression in single-cell transcriptomic conditions (Liu and Shomron, 2021), there are no data reported for such analysis in the retina. However, systematic analysis of miRNA expression and variability in the mouse (Soundara Pandi et al., 2013) and human neural retina and RPE/choroid tissues (Karali et al., 2016) have been reported. Interestingly, among the top 30 expressed miRNAs in retina are reported several miRNAs that present a role in mitochondrial-mediated eye diseases (i.e., miR-181a/b, miR-182, miR-183, miR-204, let-7a, miR-9, miR-96, miR-125b, miR-100, and miR-181c; see Table 1). Notably, many of the miRNAs here described and associated with mitochondria-mediated eye diseases can be classified as MitomiR (Purohit and Saini, 2021) (Table 1) since they regulate important transcripts impacting different mitochondrial pathways (Table 2), thus suggesting an additional possible role of these miRNAs in the pathogenesis and therapy of these disorders.

**TABLE 2:** Mitochondrial-related targets and pathways modulated by MitomiR.

**MiRNA**	**Targets**	**Mitochondria-related pathways**	**References**
MiR-8/miR-200 family	MFF	Mitochondrial dynamics	Eades et al., 2011; Zhu et al., 2012; Yao et al., 2014; Lee et al., 2017
	TFAM	Mitochondrial biogenesis	
	KEAP1	Oxidative stress response	
	BCL2 and XIAP	Mitochondria-mediated apoptosis	
MiR-9	BCL2L11	Mitochondria-mediated apoptosis	Wei et al., 2016
MiR-16	BCL2	Mitochondria-mediated apoptosis	Cimmino et al., 2005; Nishi et al., 2010
	ARL2	Mitochondrial ADP/ATP	
MiR-17	SOD2, TRXR2, and GPX2	Antioxidant response	Xu et al., 2010; Weng et al., 2014; Lu et al., 2016
	BIM-S	Mitochondria-mediated apoptosis	
	MFN2	Mitochondrial dynamics	
MiR-19a	PTEN	Mitochondria-mediated apoptosis	Zhao et al., 2017
MiR-21	BCL2	Mitochondria-mediated apoptosis	Dong et al., 2011
MiR-25	MCU	Mitochondrial Ca^2+^ uptake	Zhang et al., 2012; Marchi et al., 2013; Wu et al., 2015, 2017; Feng et al., 2016
	MOAP1; PTEN; BIM	Mitochondria-mediated apoptosis	
	NCOA3	Release of mitochondrial DNA	
MiR-27	PHB	Mitochondrial dynamics	Kang et al., 2013; Kim et al., 2016; Shen et al., 2016; Li H. et al., 2017
	PINK	Mitophagy	
	FOXJ3	Mitochondrial biogenesis	
	BAX	Mitochondria-mediated apoptosis	
MiR-29 family	MCL 1 and BAX	Mitochondria-mediated apoptosis	Mott et al., 2007; Garzon et al., 2009; Xue et al., 2016; Muluhngwi et al., 2017; Caravia et al., 2018; Jing et al., 2018
	PGC1α	Mitochondrial biogenesis	
	ATP5G1 and ATPIF1	Mitochondrial bioenergetics	
MiR-31	SIRT3	Oxidative stress response	Lee et al., 2016; Kao et al., 2019
	SDHA	Mitochondrial metabolism	
MiR-34a/c	BMF; CYC	Mitochondria-mediated apoptosis	Catuogno et al., 2013; Hu et al., 2020
	TXNRD2; SOD2	Antioxidant response	Bai et al., 2011; Fan et al., 2017; Zhu et al., 2017; Thounaojam et al., 2019
	SIRT1	Mitochondrial biogenesis	
	Notch2	Mitochondria-mediated apoptosis	
MiR-96	CASP9	Mitochondria-mediated apoptosis	Iwai et al., 2018
MiR-106b	MFN2	Mitochondrial dynamics	Wu H. et al., 2016; Li P. et al., 2017; Xu et al., 2017; Zhang C. et al., 2021
	OPTN, MFN2, and NDP52	Mitophagy	
	MCL1; DR4	Mitochondria-mediated apoptosis	
MiR-125b	BIK	Mitochondrial metabolism	Xie et al., 2015; Duroux-Richard et al., 2016; Hu et al., 2018
	MTP18	Mitochondrial dynamics	
	MCL1; HAX1	Mitochondria-mediated apoptosis	
MiR-145	BNIP3	Mitochondria-mediated apoptosis	Li et al., 2012
MiR-146a	CypD	Mitochondria-mediated apoptosis	Su et al., 2021; Heggermont et al., 2017
	DLST	Oxidative metabolism	
MiR-149	PARP-2	NAD^+^ content and mitochondrial biogenesis	Mohamed et al., 2014
MiR-155	TFAM	Mitochondrial biogenesis	Quinones-Lombrana and Blanco, 2015; Tsujimoto et al., 2020
	BAG5	Mitophagy	
MiR-181a/b/c	PINK1 and Parkin	Mitophagy	Cheng et al., 2016
			Indrieri et al., 2019
			Das et al., 2012; Ouyang et al., 2012; Rivetti di Val Cervo et al., 2012; Wang L. et al., 2014; Wang et al., 2015; Barbato et al., 2021
	BCL2, MCL1, BCL2L11, and XIAP	Mitochondria-mediated apoptosis	
	SIRT1, TFAM, and NRF1	Mitochondrial biogenesis	
	MT-COI, COX11, and COQ10B	OXPHOS	
	SIRT1 and PRDX3	Antioxidant response	
	GPX1	Oxidative stress	
MiR-183	IDH2	TCA cycle	Tanaka et al., 2013
MiR-195	MICU1	Mitochondrial Ca^2+^ uptake	Singh and Saini, 2012; Zhou et al., 2013; Zhang et al., 2018; Rao et al., 2020
	ARL2	Mitochondria-mediated apoptosis	
	MFN2	Mitochondrial dynamics	
	BCL2	Mitochondria-mediated apoptosis	
	SIRT3	Mitochondrial energy metabolism	
MiR-204	PGC1a	Mitochondrial biogenesis	Hwang et al., 2016; Houzelle et al., 2020; Zhang L. et al., 2021
	BCL2	Mitochondria-mediated apoptosis	
	TRPML1	Mitophagy and ROS production	
MiR-383	PRDX3	Antioxidant response	Li et al., 2013
MiR-497	BCL2	Mitochondria-mediated apoptosis	Yadav et al., 2011; Wu R. et al., 2016

*TCA, tricarboxylic acid; ROS, reactive oxygen species.*

## Conclusion

MicroRNAs are promising therapeutic tools due to their capability to simultaneously modulate multiple pathways involved in disease pathogenesis and progression. Moreover, they also represent a class of interesting molecules useful as disease predictive/prognostic biomarkers. Indeed, several miRNAs (let-7a, miR-450, miR-107, miR-204, miR-21, and miR-149 for glaucoma; miR-17 and miR-125b for AMD; miR-126, miR-146a, miR-155, miR-132, miR-21, and miR-34a/c for DR) differentially expressed in body fluids (i.e., serum, plasma, and vitreous liquid or tears) of eye diseases associated with mitochondria dysfunctions (EDAMDs) human patients may be already considered as clinically relevant biomarkers (bold miRNAs in Table 1).

Recently, an increasing interest is growing about MitomiRs, which regulate mitochondrial function. As described before, many MitomiRs have been linked to mitochondria-mediated eye diseases, including both rare PMEDs and common retinal diseases (Tables 1, 2). Due to the genetic heterogeneity that characterizes PMEDs and to the big complexity that underlies the most common retinal disorders (e.g., glaucoma, AMD, and DR), no effective treatments are still available. For the above-mentioned reasons, miRNA-based gene/mutation-independent therapeutic strategies may represent a great promise. By targeting common dysregulated pathways that play a key effector role in retinal damage (e.g., mitochondrial dysfunction, oxidative stress, inflammation, and neovascularization), miRNA modulation can protect retinal cells regardless of the primary etiology of the addressed disorder. Considering that the retina is an easily accessible tissue, we believe that the potential application of miRNA therapeutics in retinal disorders could rapidly move to the clinic.

## Author Contributions

SC and AI conceived the study. SC, FM, and AI wrote the manuscript. All authors contributed to the article and approved the submitted version.

## Conflict of Interest

The authors declare that the research was conducted in the absence of any commercial or financial relationships that could be construed as a potential conflict of interest.
